# Work-Family Conflict, Perceived Organizational Support and Professional Commitment: A Mediation Mechanism for Chinese Project Professionals

**DOI:** 10.3390/ijerph15020344

**Published:** 2018-02-15

**Authors:** Junwei Zheng, Guangdong Wu

**Affiliations:** 1 Faculty of Civil Engineering and Mechanics, Kunming University of Science and Technology, Kunming 650500, China; zjw1989@kmust.edu.cn; 2 Department of Construction Management, Jiangxi University of Finance and Economics, Nanchang 330013, China

**Keywords:** work-family conflict, perceived organizational support, professional commitment, construction enterprises, structural equation modeling, bootstrapping

## Abstract

Projects are characterized by long working hours, complex tasks and being a kind of temporary organization. As such, work-family conflict is particularly prominent for project employees. This research examined whether and how work-family conflict affects professional commitment among Chinese project professionals. Research hypotheses were developed to explore the relationship between work-family conflict, professional commitment to the project and the mediating effects of perceived organizational support. Data were collected from 327 project managers or professionals working in construction enterprises in China; data were analyzed using structural equation modeling, applying the bootstrapping method. Results showed that there were three dimensions of work-family conflict: time-based conflict, strain-based conflict and behavior-based conflict. There were two dimensions of perceived organizational support: emotional support and instrumental support. The study also tested the negative effect of work-family conflict on professional commitment and the positive effect of perceived organizational support on professional commitment. Specifically, time-based conflict and emotional support had positive effects on professional commitment. Perceived organizational support had a total mediating effect between work-family conflict and professional commitment. The strain-based conflict dimension of work-family conflict had negative impacts on professional commitment through perceived emotional support and instrumental support. Overall, our findings extend a better understanding of work-family conflict and professional commitment in the project setting and verify the importance of social support in balancing work and family and improving employee mobility.

## 1. Introduction

Work-family conflict has become an increasingly important topic in organizational behavior and human resource management research. This is because the work-family conflict has been verified to be correlated with the employee’s work life, family life, public health and well-being [1]. It also plays a negative role in organizational performance, commitment [2] and strategy [3]. Management and psychology scholars have paid significant attention to work-family conflict for a long time, and previous research has verified the concept and influencing factors of work-family conflict based on the role theory perspective [4]. Ng and Reldman [5] highlighted the curvilinear relationship between hours worked and work-family conflict variables based on the social identity perspective. Powell and Greenhaus [6,7] found that individual characteristics (e.g., gender role) lead to the interaction of the work-family interface. Hence, the work-family conflict has become a common research topic at the individual and organizational level.

There has been increasing research attention on work-family conflict issues in the project context. For example, Lingard et al. [8] established and tested a multidimensional scale of work-family conflict in the Australian construction industry. Liu and Low [1] found that Chinese project managers experienced different types of work-family conflicts: time-based, strain-based and behavior-based conflict. Francis et al. [9] compared the work-family experience of the public sector and private sector employees. Bowen et al. [10] examined the relationship between the antecedents (e.g., work contact), work-family conflict and consequent outcomes of psychological distress and sleep problems in a sample of South African construction professionals. Turner and Mariani [11] explored how project managers managed their work-family interface. Xia et al. [12] examined the relationships between work-family conflict, project commitment and project citizenship behavior. Most studies to date have focused on the question of whether there is work-family conflict among project managers or employees in the construction industry and how to measure the work-family conflict variables for project employees in that specific context. 

Thus, previous studies have identified work characteristics of construction projects, including long working hours, inflexible duration and complex tasks [1,13], which could likely lead to the high level of work-family conflict experienced by project managers or employees [12]. In the labor-intensive context of the construction industry, features such as high risk [14] and heavy workload increase the work overload and create high levels of work-family conflict [15]. In temporary and dynamic project-based organizations, high work-family conflict felt by project managers may influence the work outcomes [16] and project commitment [17]. A career commitment to projects indicates that employees want to do what is needed to achieve project targets [18]. Furthermore, in the traditional Chinese culture, obtaining a career is a family expectation. As such, work would contribute to the family [1,19]. The career commitment for the organization represents a reciprocal act to achieve work-family balance [20]. The effects of work-family conflict on commitment have been tested in organization management research [21]. The relationship between work-family conflict and career satisfaction or organizational commitment has been the focus of organization management studies [20,21,22]. Although existing studies have focused on work-family issues of individuals in construction project settings [1,10,11,12,23], few studies have specifically investigated organizational commitment and professional commitment with respect to project management professionals and project workers [24,25], and there is less research about the inner mechanisms of work-family conflict and commitment in construction project settings. In summary, studies have investigated either the experience of work-family conflict or the professional commitment of project managers, professionals or other employees. However, little is known about whether and how work-family conflict and professional commitment connect with one other. 

Meanwhile, a few studies examined the negative relationship between work-family conflict and perceived organizational support [26], and less research focused on why employees with high levels of work-family conflict would have low levels of perceived organizational support [27]. Although work-family conflict and perceived organizational support were separately related to employee job attitudes or behaviors, the process of how work-family conflict undermined the social exchange relationship between employee and the employing organization was not clear. Moreover, existing research has indicated that work-family conflict was negatively associated with job attitudes, and perceived organizational support was positively associated with job attitudes, while fewer studies examined the social exchange process of linking work-family conflict and job attitudes [27,28,29]. Thus, whether the effect of work-family conflict on employee job attitudes (e.g., organizational commitment, professional commitment) was indirect, it was mediated through the social exchanges construct of perceived organizational support. Therefore, the mediating role of perceived organizational support between work-family conflict and job attitudes (i.e., professional commitment) was proposed in this study.

The goal of this study is to explore how to balance work-family problem in the project context, in order to motivate project employees to achieve project targets and success, which are beyond the insights offered by more traditional models of work-family conflict. First, using an inter-role conflict theoretical perspective [30], our study addresses this research by examining the effect of work-family conflict felt by project employees on professional commitment. To obtain an in-depth understanding of the effect, we also unveiled the specific forms of work-family conflict of project employees, including time-based conflict, strain-based conflict and behavior-based conflict. Next, based on the previous study on the effect of supervisor support on employee conflict [8] and combining this with the social support theoretical perspective [31], we propose that the perceived organizational support will alleviate the work-family conflict problem and promote project employee commitment in the context of construction projects. Hence, the indirect effect of work-family conflict on professional commitment via perceived organizational support is also examined to indicate the inner mechanism. 

Our findings contribute to theory and practice in several ways. First, the study reveals that work-family conflict negatively influences project employees’ professional commitment. While the project management literature has emphasized the importance of project success, little is known about what indicators negatively influence employee behavior and project target achievement. Second, previous studies have examined the extent to which the indirect effect of work-family conflict on organizational commitment depends on perceived supervisor support [32]. This study unveils yet another indirect effect of work-family conflict on project professional commitment, through the mediator of perceived organizational support. Study findings demonstrate that perceived organizational support may exert both a moderating and mediating effect. Thus, this study provides a more integrative view of how project managers or employees may decrease work-family conflict and promote professional commitment, by gaining organizational esteem and concern and improving relationships with supervisors.

This paper organized as follows. First, it presents a detailed review of the concepts of work-family conflict, professional commitment and perceived organizational support. The paper then proposes the hypotheses to be tested. Second, the paper presents the sampling procedure, the measures used in the survey scales and the research methodology. Third, the measurement model analysis and the findings of the structural equation modeling with bootstrapping are explained. Finally, the paper closes with a discussion, implications and future research directions.

## 2. Theory and Hypotheses

### 2.1. Work-Family Conflict 

Work-family conflict refers to a form of inter-role conflict, in which the role pressures from the work and family domains are mutually incompatible in some way [4,33]. There are three major forms of work-family conflict based on this definition: (i) time-based conflict; (ii) strain-based conflict; and (iii) behavior-based conflict. Time-based conflict may occur when time devoted to one role makes it difficult to participate in another role [33]. This form of conflict is consistent with excessive work time and schedule conflict [34], as well as role overload [30]. Strain-based conflict suggests that strain experienced in one role limits the ability to meet the demands of another role, or interferes with participation in another role [35]. Behavior-based conflict occurs when the behaviors required in one role are counterproductive in another role [4].

Given the temporary and dynamic nature of projects, all three forms of work-family conflict are experienced by project employees [12]. First, project employees must address different problems and uncertainties, due to the task-based characteristics of complexity, multi-participant, high-risk and peak work load [13,36]. This results in having insufficient time to meet family demands. Then, time-based conflicts occur. Second, because of the project complexity associated with large budgets, the nonlinear relationship, irregular resource allocation and high uncertainty [37], project employees, especially leaders or managers, must bear heavy responsibilities, balance the demands of many stakeholders and rapidly adjust to the dynamic requirements [38]. Hence, project managers or professionals experience work overload, causing stress [39] and likely leading to strain-based conflict. Finally, considering the dynamic and uncertain project environment, project managers or professionals experience challenging tasks, generating bad emotions (e.g., frustration, disturbance) [37,38]. Moreover, the negative emotion and the project-based context may influence project managers’ work behavior and personality, impacting the project’s success [40]. Thus, project employees must control and regulate their emotions and behaviors to achieve project success, but they may show a different demeanor with family members. The difference in emotion and behavior between the work interface and family interface may lead to behavior-based conflict [12].

Besides, according to Gutek et al. [41], work-family conflict can be distinguished as two components, i.e., work interference with family (WIF) and family interference with work (FIW). Because of construction projects’ characteristics of long work hours, inflexible scheduling and complex task [1,12,13], as well as too many hours worked per week, the inflexibility of the task schedule would raise work-family conflict [27], and project employees never have enough time to spend with family. Thus, the work interference with family would be more prominent than the family interference with work in the construction project context, and the studied variable of work-family conflict in this study was mainly concerned with the interference of work with family.

### 2.2. Project Professional Commitment

Professional commitment refers to the kind of work commitment that focuses on the importance of a career in one’s life [42]. Professional commitment is an important indicator determining employees’ work behavior [43] and indicates an individual’s attitude toward the chosen profession [44]. The definition of professional commitment is equivalent to career commitment [45] or occupational commitment. Projects have job features such as long working hours, inflexible schedules and complex tasks [12,46]; as such, project employees must dedicate significant time, energy, knowledge and skills, leading to their commitment to the profession [24,47]. Moreover, given the temporary nature of projects, professional commitment benefits project employees in doing well from project to project and complements organizational commitment [48].

### 2.3. Perceived Organizational Support

Perceived organizational support has been defined as “employees’ beliefs about the degree to which the organization views their contributions and has concern for their well-being” [49]. This perceived organizational support promotes the employees’ sense of obligation to increase their commitment to the organization and achieve organizational goals [49]; this form of support also leads to positive work attitude and performance [50] and positively influences mental health [51]. Previous research has examined the role of perceived organizational support in balancing the work-family relationship [52]. Perceived organizational support provides a buffering effect on work-family conflict [53] and weakens the influence of work-family conflict on work outcomes [54]. Studies have considered the effect of perceived support on a sample of professional managers [55] and nursing professionals [56]. However, little research has explored the effects of perceived support from the temporary organization on project professionals.

### 2.4. Work-Family Conflict and Professional Commitment

Past research suggests that work-family conflicts significantly affect career commitment [57]. Work-family conflict is an important variable in the stressor domain and may be higher in contemporary work settings [58] (e.g., contemporary projects), due to the long working hours, changing demands and stress arising from complex tasks [59]. Rising work demands from the project environment may limit a project employee’s ability to effectively complete both the work and family roles; as such, work-family conflict may negatively influence career commitment [58]. When work roles spill over into family roles, less time is devoted to the career, and more work-family conflict is introduced, affecting career commitment [60]. Studies have examined the relationship between work-family conflict and organizational commitment [32,61]. However, the influence of work-family conflict on professional or career commitment has not received adequate empirical focus. As such, the following hypotheses were developed.

**Hypothesis** **1 (H1).** Work-family conflict is negatively related to the professional commitment of project employees.

**Hypothesis** **1a (H1a).** Time-based conflict is negatively related to the professional commitment of project employees.

**Hypothesis** **1b (H1b).** Strain-based conflict is negatively related to the professional commitment of project employees.

**Hypothesis** **1c (H1c).** Behavior-based conflict is negatively related to the professional commitment of project employees.

### 2.5. Perceived Organizational Support and Project Professional Commitment

Previous studies have found that perceived support positively relates to organizational commitment (e.g., affective commitment [62]). According to the positive relationship between perceived organizational support and job satisfaction [50], we can assume that perceived organizational support relates to the professional commitment of employees. A meta-analysis suggested that perceived organizational support plays a positive role in employees’ commitment [63]. Specifically, Kim [64] found that perceived organizational support was the antecedent variable of commitment and satisfaction for a sample of sports officials. Because emotional support and instrumental support represent agents of the organization [49,65], the perceived organizational support may promote positive attitudes and behavior toward the organization in general [32]. Consistent with this viewpoint, perceived organizational support would positively relate to professional commitment. These led to the following hypotheses.

**Hypothesis** **2 (H2).** Perceived organizational support is positively related to the professional commitment of project employees.

**Hypothesis** **2a (H2a).** Emotional support is positively related to the professional commitment of project employees.

**Hypothesis** **2b (H2b).** Instrumental support is positively related to the professional commitment of project employees.

### 2.6. Mediating Role of Perceived Organizational Support

Previous research found that the correlation between work-family conflict and organizational commitment is a stressor-strain relationship; perceived organizational support plays a buffering role in the stressor-strain relationship [66]. According to social exchange theory [67], employees who experience concern and good treatment from an organizations may express a reciprocal attitude and behavior toward their employer and organization [32,68]. According to the norm of reciprocity [69], employees who obtain a high perceived organizational support may establish their satisfaction and transfer it to a feeling of psychological attachment to the organization, as well as may respond favorably to the organization in the form of positive job attitudes or behaviors. Individuals may build a social exchange relationship with the organization as a whole [70] and build trustful social relationships with mutual loyalty [71]. The theory of social exchange and the norm of reciprocity could explain the relationship between perceived organizational support and job attitudes. Furthermore, work-family conflict could decrease the quality of perceived organizational support, and employees would reciprocate unfavorable treatment with negative job attitudes and behaviors [72]. The buffering effects of perceived organizational support have been found studying Western samples [73], Brazilians [32] and different professionals (e.g., doctors [51], migrant workers [74], academics [75]). Therefore, we assume that the effect of work-family conflict on job attitudes (i.e., professional commitment) is indirect via perceived organizational support in project professional samples. These led to the following hypotheses.

**Hypothesis** **3 (H3).** Work-family conflict has a negative indirect effect on professional commitment via perceived organizational support.

**Hypothesis** **3a (H3a).** Time-based conflict has a negative indirect effect on professional commitment via emotional support and instrumental support.

**Hypothesis** **3b (H3b).** Strain-based conflict has a negative indirect effect on professional commitment via emotional support and instrumental support.

**Hypothesis** **3c (H3c).** Behavior-based conflict has a negative indirect effect on professional commitment via emotional support and instrumental support.

**Figure 1:**
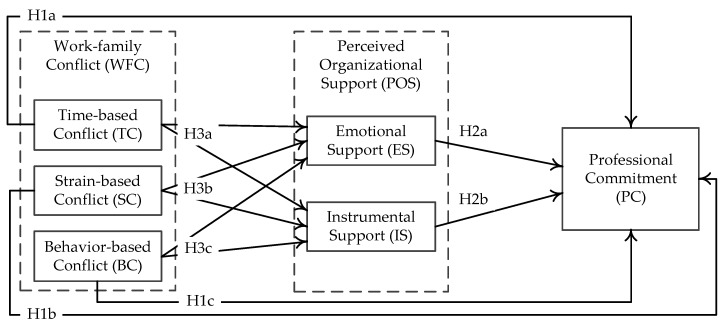
The conceptual model.

Figure 1 shows the theoretical framework integrating all proposed hypotheses in this study.

## 3. Methods

### 3.1. Sample and Data Collection

This study was conducted with employees of construction companies in China. We distributed the surveys along with the channels of on-line and e-mail to assure confidentiality and voluntary participation. We briefed the participants about the goal of this study and emphasized that the company or others would not have access to their answers or any information. To better protect the confidentiality of participants, we distributed our surveys to different construction projects of different companies. A total of 346 employees participated, including managers (e.g., project manager) and engineering technicians (e.g., quality inspector), who completed and returned the questionnaires. In order to ensure the authenticity of the responses, the IP address and the attribution associated with the on-line survey was checked so that the majority of responses were not from one location or a single construction project’s site. Thus, of the 346 total responses, 327 responses were valid, and thus, the response rate was much higher than the norm of 20–30% in most construction studies [76]. Table 1 provides the profile of respondents.

### 3.2. Measures

This study improved the scales based on established scales from existing literature, combined with the context associated with the Chinese construction enterprise. Then, scale development procedures were followed based on the methodology suggested by Churchill [77]. This included an exploratory factor analysis and confirmatory factor analysis, reliability test, discriminant validity test, and others. The final items of the studied constructs were developed by making minor modifications, based on subsequent reliability and validity testing. All scales were constructed using a five-point Likert type scale (“1” = strongly disagree, “5” = strongly agree).

#### 3.2.1. Work-Family Conflict

The 10 items developed by Carlson et al. [33] and Stehpens and Soomer [78] were used to measure work-family conflict. Reliability for all items, measured using the alpha coefficient, was 0.828. The 10 items were divided into three dimensions: time-based work-family conflict (e.g., “my work keeps me from my family activities more than I would like”, 4 items), strain-based work-family conflict (e.g., “tension and anxiety from my family life often weakens my ability to do my job”, 4 items) and behavior-based work-family conflict (e.g., “the behaviors that work for me at home do not seem to be effective at work”, 2 items). The reliabilities were 0.703, 0.723 and 0.808, respectively.

#### 3.2.2. Perceived Organizational Support

Three-dimensional scales developed by Eisenberger et al. [79,80] and Rhoades et al. [81] were used to measure perceived organizational support, including emotional support (e.g., “my organization really cares about my well-being”, 4 items) and instrumental support (e.g., “my organization allows me to work at home on family problems”, 3 items). The reliabilities were 0.823 and 0.800, respectively, and the reliabilities of all 7 items scales were 0.815.

#### 3.2.3. Professional Commitment

Based on Gary [44], five items were generated to measure professional commitment; for example, “if had all the money needed, I would still work in the current career” and “ideal vocation too well to give it up”. The reliability coefficient was 0.677.

#### 3.2.4. Control Variables

Two types of variables were controlled: first, the enterprise characteristics that might influence employee behaviors, including staff size; second, the employees’ demographic characteristics, which were statistically controlled. These included gender, age, working years and career type. These factors were controlled due to their potential effects on the employees’ professional commitment and relationships with supervisors and family members.

### 3.3. Analysis

Prior to assessing the measurement, all studied variables were collected from the same source; as such, we needed to test the common method variance. Harman’s one-factor test was conducted [82], whereby all items were simultaneously entered into the factor analysis, using a principal component analysis with a varimax rotation. The results concluded that more than one factor explained 54.45% of the variance, with the first factor accounting for 24.23% of the total variance. This indicates that no single factor structure emerged, and no one factor accounted for most of the total variance. In addition, according to Podsakoff et al. [83], a series of analyses should be conducted to check for the presence of common method variance or common method bias. Thus, another common method variance test, suggested by Malhotra et al. [84], was conducted. The fit indexes indicated that the model consisting of a single latent variable has a very poor fit, such as χ^2^/*df* = 8.185, Comparative Fit Index (CFI) = 0.418, Tucker–Lewis Index (TLI) = 0.357 and RMSEA = 0.148. Hence, the common methods’ bias was not a significant problem with the collected dataset.

Before testing the hypotheses, the exploratory factor analysis and confirmatory factor analysis were conducted to assess the measurement model comprised by the studied variables. The exploratory factor analysis with principle component analysis and varimax rotation was conducted to check the unidimensionality for the adopted scales [85], which used SPSS Version 22.0 (Armonk, NY, USA). The confirmatory factor analysis was conducted to examine the fit index of factor models [85], using AMOS Version 24.0 (Armonk, NY, USA). Then, the reliability test was analyzed using Cronbach’s α coefficient and composite reliability. The convergent validation was evaluated using Average Variance Extraction (AVE), and the discriminant validation was analyzed by comparing the square root of AVE values and correlation coefficients.

The Structural Equation Modeling (SEM) technique has been widely used in construction management studies [86,87,88] and thus was conducted to test the hypotheses, using MPLUS Version 7.0 (Los Angeles, CA, USA). More specifically, a default model was established first to analyze the total effects of perceived organizational support and work-family conflict on professional commitment. Then, the indirect effects of perceived organizational support on professional commitment via work-family conflict were accounted for using the Confidence Interval (CI), computed using the bootstrapping method. This method was more powerful than the Sobel test or Baron and Kenny approach to test the mediation effects [89,90]. The bootstrapping process was repeated at least 1000 times to generate the 95% confidence interval. If zero was not found between the lower and upper bound, the results associated with the indirect effect was accepted as rejecting the null hypothesis, with a *p*-value less than 0.05 [91].

## 4. Results

### 4.1. Exploratory Factor Analysis (EFA) and Item Reduction

Exploratory factor analysis was used to determine the factor structure of three distinct factors related to the work-family conflict. A factor structure for the three dimensions of perceived organizational support was also identified. When analyzing the single factor structure of professional commitment, one item (i.e., PC 1) with a loading of less than 0.50 was found. After deleting the small loading item, the EFA results demonstrated a three-dimensional structure for work-family conflict ((i) time-based work-family conflict, 4 items; (ii) strain-based work-family conflict, 4 items; (iii) behavior-based work-family conflict, 2 items). There was also a two-dimensional structure for perceived organizational support ((i) emotional support, 4 items; (ii) instrumental support, 3 items); and a single dimension for professional commitment (4 items). These dimensions are shown in Table 2. Table 2 also provides the percentage of variance explained by the different constructs and the alpha coefficients of different factors. 

### 4.2. Confirmatory Factor Analysis and Item Design Assessment 

The measurement models in the study were defined as reflective models [92,93], and the studied variables had different dimensions. Then, a first-order confirmatory factor analysis and second-order confirmatory factor analysis were conducted. The observed variables or items were reflective indicators of each dimension or latent variable (e.g., time-based work-family conflict and emotional support). The dimensions (first-order factors) were the reflective indicators of the latent second-order construct (e.g., work-family conflict and perceived organizational support). 

Table 3 shows the fit statistics for the structural models of the studied constructs. When considering the constructs of Work-Family Conflict (WFC) and Perceived Organizational Support (POS), the second-order model and first-order model were the same fit. The second-order factor model was not significantly better than the first-order three-factor model. The two models are mathematically equivalent [94]. Nevertheless, the second-order model allowed for co-variation among first-order factors, by accounting for the corrected errors that were common in first-order confirmatory factor analysis [95]. As such, there were latent second-order variables, such as work-family conflict and perceived organizational support; the two constructs had separate different dimensions. The Composite of Reliabilities (C.R.) for different dimensions were above 0.70, and the Average Variances Extraction (AVE) were mostly close to or reached the criterion of 0.50. This indicated that the variances captured by the construct were larger than the variances caused by measurement error [96]. 

### 4.3. Discriminant and Convergent Validation

The fit indexes were used to compare the alternative models. Table 4 shows that the seven-factor model was significantly better than the competing models. For example, CFI (Comparative Fit Index) and TLI (Tucker–Lewis Index) were close to the criterion of 0.90 [97,98]; the RMSEA (Root Mean Square Error of Approximation) was close to 0.08 as an indicator of good fit [99]; and SRMR (Standardized Root Mean square Residual) was lower than 0.05 as an indicator of good fit [98]. 

The correlation coefficients among the seven factors ranged from −0.188–0.566, as listed in Table 5. The square roots of AVE (from 0.621–0.832) were larger than the correlation coefficients of the seven factors (the maximum value was 0.566), indicating the discriminant validity of the seven factors [96]. Combined with the significance of the correlation coefficients among the seven factors and the seven-factor model fit of CFA analysis, the results demonstrated the good convergent validity of the observed items for the factor structure.

### 4.4. Independent Samples t-Test

The independent samples *t*-test with bootstrap (*n* = 1000, 95% CI) was conducted using SPSS Version 22.0 (Armonk, NY, USA). As shown in Table 6, For work-family conflict, there was a significant difference between scores for the female group (mean = 3.488, S.D. = 0.520) and scores for the male group (mean = 2.584, S.D. = 0.619), *t* = 13.863, *p* < 0.001 (two-tailed test). For perceived organizational support, there was no significant difference between scores for the female group (mean = 3.160, S.D. = 0.7844) and scores for the male group (mean = 3.309, S.D. = 0.753), *t* = −1.661, *p* = 0.098 > 0.05 (two-tailed test). For professional commitment, there was no significant difference between scores for the female group (mean = 3.454, S.D. = 0.995) and scores for the male group (mean = 3.644, S.D. = 0.679), *t* = −1.791, *p* = 0.075 > 0.05 (two-tailed test). Therefore, there was a gender difference in work-family conflict for project employees.

### 4.5. Structural Equation Modeling

#### 4.5.1. The Direct Effects of Work-Family Conflict and Perceived Organizational Support

Table 7 shows the main direct effect of work-family conflict or perceived organizational support on professional commitment, using the software MPLUS Version 7.0 (Los Angeles, CA, USA). The table shows that the main effect of work-family conflict on professional commitment was negative and significant (β_WFC→PC_ = −0.288, *p* < 0.01, Model 1). This result supports Hypothesis 1; Appendix A provides the syntax of Model 1 with MPLUS. Specifically, to test the indirect effects of the dimensions of work-family conflict on professional commitment, we computed estimates for three paths in Model 2: time-based conflict → professional commitment (β_TC→PC_ = −0.401, *p* < 0.05), strain-based conflict → professional commitment (β_SC→PC_ = 0.157, *p* > 0.05) and behavior-based conflict → professional commitment (β_BC→PC_ = −0.050, *p* > 0.05). This set of analyses supports Hypothesis 1a, but not Hypotheses 1b and 1c. 

The estimate of the direct effect of perceived organizational support on professional commitment was positive and significant in Table 7 (β_POS→PC_ = 0.815, *p* < 0.001, Model 3), which provides support for Hypothesis 2. To test the effects of the dimensions of perceived organizational support on professional commitment, the three paths were also estimated in Model 4: emotional support → professional commitment (β_ES→PC_ = 0.525, *p* < 0.001) and instrumental support → professional commitment (β_IS→PC_ = 0.120, *p* < 0.05). These outcomes support Hypotheses 2a and 2b. 

Most fit indices of the tested models in Table 7 almost fulfilled the requirements. Moreover, the model fit indices for the effects from perceived organizational support to professional commitment (CFI, TLI > 0.90; RMSEA < 0.08) were better than the indices for the effects from work-family conflict to perceived organizational support (CFI, TLI close to 0.90; RMSEA close to 0.08). The relationship between perceived organizational support and professional commitment was more significant than the correlation between work-family conflict and perceived organizational support.

#### 4.5.2. The Mediation Effect of Perceived Organizational Support

Hypothesis 3, which predicted the mediation effect of perceived organizational support on the link between work-family conflict and professional commitment, was tested using the software MPLUS Version 7.0 (Los Angeles, CA, USA). The indirect effects were computed using Bias-Corrected Confidence Intervals (BC CI); Appendix B lists the syntax. The estimate of the indirect effect was computed as a product of three paths in Table 8: work-family conflict → perceived organizational support (β_WFC→POS_ = −0.247, 95% BC CI (−0.524, −0.056), range does not include zero, significant); perceived organizational support → professional commitment (β_POS→PC_ = 0.826, 95% BC CI (0.517, 1.261), range does not include zero, significant); and work-family conflict → professional commitment (β_WFC→PC_ = −0.054, 95% BC CI (−0.252, 0.190), range includes zero, not significant). Therefore, the effect of work-family conflict on professional commitment was not significant when perceived organizational support was also included in the model. The direct effect of work-family conflict on professional commitment remained non-significant when the mediator of perceived organizational support was in the model. This suggests that perceived organizational support totally mediated the effect of work-family conflict on professional commitment. The fit indices in Table 8 also met the requirements (CFI, TLI close to 0.90; RMSEA < 0.08). Therefore, the results partially support Hypothesis 3.

In addition, we conducted the analysis separately according to the male sample (*n* = 219) and female sample (*n* = 108). As shown in Table 8, the indirect effect in the male sample was significant (95% BI CI (−0.645; −0.124), range does not include zero). However, the mediation effect analysis in the female sample cannot be converged, so the results have not been indicated in the paper. The number of female samples was small such that the bootstrap or variance-based structural equation modeling cannot successfully iterate. Thus, there was a significant difference between male and female when work-family conflict influences professional commitment via perceived organizational support.

Hypotheses 3a, 3b and 3c predicted that emotional support and instrumental support mediated the indirect effects of time-based conflict, strain-based conflict or behavior-based conflict on professional commitment. We tested these three hypotheses using the bootstrapping approach [91]. The results in Table 9 show the specific mediation effect. The path from strain-based conflict to professional commitment via emotional support was negative and significant (−0.079, 95% BC CI (−0.205, −0.011), range does not include zero). The path from behavior-based conflict to professional commitment via instrumental support was negative and significant (−0.044, 95% BC CI (−0.114, −0.009), range does not include zero). These results demonstrated that the mediating effects of perceived organizational support between strain-based conflict and professional commitment (i.e., Hypothesis 3b) were supported. Besides, the paths from time-based conflict to professional commitment via emotional support or instrumental support were negative and non-significant (−0.093, 95% BC CI (−0.236, 0.010), range includes zero; −0.040, 95% BC CI (−0.129, 0.000), range includes zero). The paths from behavior-based conflict to professional commitment via emotional support or instrumental support were negative and non-significant (−0.057, 95% BC CI (−0.142, 0.014), range includes zero; −0.011, 95% BC CI (−0.058, 0.016), range includes zero). These results do not support Hypothesis 3a nor Hypothesis 3c.

**Figure 2:**
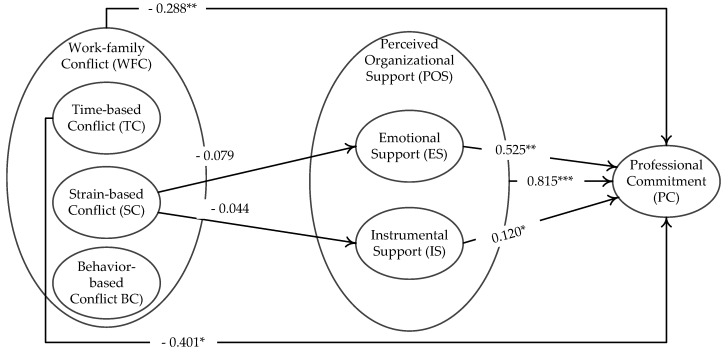
The hypothesis testing results of the theoretical model. Notes: the coefficients above the single arrow lines indicate the significant indirect effects. *** *p* < 0.001; ** *p* < 0.01, * *p* < 0.05.

Figure 2 plots the results of hypotheses testing. Table 10 summarizes the results. 

## 5. Discussion

### 5.1. Effects of Work-Family Conflict on Professional Commitment

This study delineated whether and how work-family conflict affects the professional commitment of Chinese project professionals. Analyses revealed that work-family conflict had a negative effect on project professional commitment; time-based conflict was the main influence on professional commitment. Consistent with previous research of the relationship between work-family conflict and organizational commitment [21], the work-family conflict of project professionals was also negatively related to commitment to the project profession. However, we did not examine the assumptions that strain-based conflict and behavior-based conflict negatively affect professional commitment. Because of the project characteristics of long working hours and complex tasks [1,12], there was insufficient time for project employees to stay with their family members. The dimension of time-based conflict was particularly clear for Chinese project employees and reduced their project professional commitment.

### 5.2. Effects of Perceived Organizational Support on Professional Commitment

This study also found that perceived organizational support was positively associated with professional commitment. Specifically, the emotional support and instrumental support had positive effects on professional commitment. Perceived emotional support may provide psychological assistance; perceived instrumental support focused on providing actual support or aid for employees [100]. China has a culture of collectivism, a hierarchical structure and paternalistic leadership. As such, perceived emotional support and instrumental support are likely to be important to employees in the project context, who wanted more care and guidance [14,101]. Hence, increased concern and support from organizations created a more positive relationship between perceived organizational support and professional commitment in the Chinese project setting.

### 5.3. Mediating Effects of Perceived Organizational Support

The bootstrapping results indicate that perceived organizational support played a total mediation effect between work-family conflict and professional commitment. This differs from the moderating effect of perceived organizational support [32]. This study provided additional empirical evidence to support the buffering effect of social support. Moreover, perceived emotional support and perceived instrumental support played the mediating role mainly between the strain-based conflict dimension of work-family conflict and professional commitment. An effective supervisory mechanism is lacking in China [102], and there is a collective culture and high power distance in China. As such, employees are sensitive to the concerns of organizations, and organizational support is likely to be expressed as paternalistic care [101]. Because of the desire for high concern and supportive supervision, perceived emotional support and perceived instrumental support are likely to affect the relationship between work-family conflict and professional commitment.

## 6. Conclusions and Implications

### 6.1. Conclusions

This study investigated the direct effect of work-family conflict on professional commitment and the indirect effect of work-family conflict on professional commitment via perceived organizational support. Work-family conflict was found to be negatively related to professional commitment. The dimension of time-based conflict played the main negative role in affecting professional commitment beyond the dimensions of strain-based conflict and behavior-based conflict. Perceived organizational support was found to be positively related to professional commitment and the emotional support and instrumental support positively related to professional commitment. The study found that perceived organizational support had a total mediating effect between work-family conflict and professional commitment. Specifically, emotional support and instrumental support served as the mediator between strain-based conflict and professional commitment.

### 6.2. Theoretical Implications

This study contributes to our understanding of work-family conflict, perceived organizational support and professional commitment, by connecting these three constructs in the project setting. The social exchange process for linking work-family conflict and professional commitment was examined through Chinese project employees’ samples. First, this study extended work-family conflict research in the project context. The problem of work-family balance has been an increasingly important focus in the project management domain [1,15], and little research has explored the outcome variables and mechanism of work-family conflict in managing projects. For example, Xia et al. [12] examined the influence of work-family conflict on project citizenship behavior, using project commitment as the mediator. Andres et al. [103] tested the relationship between work-family conflict, satisfaction and turnover intention in the context of a military development project. This study investigated the potential effects of work-family conflict on project-related beliefs and job attitudes, i.e., perceived organizational support and project professional commitment. Results found that work-family conflict had negative effects on perceived organizational support and project professional commitment in the temporary project context; this has implications for work-family conflict in project employees’ value and attitude. 

Besides, this study focused on Chinese project professionals and considered the cultural factors. In contrast, research in Anglo culture has indicated a negative effect of work-family conflict [66]. Existing studies have acknowledged that directly extrapolating the results from Western studies to the Chinese context may cause misunderstanding [104]. This study contributes to the field by considering ways to balance the work-family problem in the Chinese project context and by examining the effects of work-family conflict on individual belief mediators (i.e., perceived organizational support) and individual attitude outcomes (i.e., professional commitment). The proposed hypotheses were partially supported, indicating the differences of work-family conflict dimensions in influencing professional commitment and the variations in the mediating effect of perceived organizational support dimensions.

### 6.3. Practical Implications

Our theoretical model has practical implications for project managers and professionals. One implication is that adopting a focus on work-family conflict will benefit project professionals. Project managers and professionals consistently experienced high levels of work-family conflict under the uncertain, complex and temporary project context [12]. Not surprisingly, handling work-family conflict is becoming an increasingly important skill for managers or leaders, and this study suggests that organizations could provide the training program for project managers or professionals to address work-family balance problems [105]. The empirical results show that time-based conflict had a primarily negative effect on professional commitment. Hence, project-based organizations should ensure project managers or professionals have enough time to spend with family members before moving from a completed project to the next project [13].

In addition, the empirical results indicated that strain-based work-family conflict affected professional commitment through perceived organizational support. The organizations in construction projects could make efforts to enhance the quality of perceived organizational support to prevent the indirect effects of work-family conflict on job attitudes (i.e., professional commitment), especially in the areas of perceived emotional support and instrumental support for the strain-based work-family conflict. Specifically, organizational supports, for example, work life-related or family life-related well-being, actual child-related support and eldercare assistance [12,106], could reduce work-family conflicts and strengthen project employees’ loyalty to the project profession. Project-based organizations should provide support to their employees, which is helpful for decreasing talent mobility and reducing the intention to leave [27], thus improving their professional commitment to the project. Meanwhile, organizations could start with the sources of work-family conflict, for example identifying the career developmental demands earlier and providing the appropriate opportunities [13], to decrease the stress of project managers or employees, thus strengthening the quality of perceived organizational support and ensuring the commitment to profession in construction projects.

### 6.4. Limitations and Directions for Future Research

This study has theoretical and methodological strengths; however, it also has limitations that highlight opportunities for meaningful future research. First, the finding that perceived organizational support mediated the effect of work-family conflict on professional commitment in the sample of project employees highlights the need to investigate additional potential mediators. For instance, social support from peers [107] or family members [108] may contribute to a buffering effect for work-family conflict. Second, the relationship between work-family conflict and professional commitment via perceived organizational support may depend on contextual factors. There were differences in demographic characteristics (e.g., gender [32], education [24]). Finally, alternative study approaches could measure different variables at different times and from different sources. This would better resolve the same-source problem and common method bias. Finally, future studies could ask different sources to rate measured variables and could conduct longitudinal research to account for the main effects of work-family conflict dimensions and the buffering effects of perceived organizational support dimensions.

## Figures and Tables

**Table 1:** Descriptive statistics of the sample.

Title	Number	Percentage	Title	Number	Percentage
Gender			Types of profession		
Male	219	66.97	Technician	7	2.14
Female	108	33.03	Construction workers	156	47.71
Total	327	100	Safety officer	73	22.32
Age			Documenter	36	11.01
30 and under	132	40.37	Quality inspector	15	4.59
31–40	156	47.71	Supervisor	36	11.01
41–50	39	11.92	Project manager	4	1.22
Total	327	100	Total	327	100
Years of working			Scale of enterprise		
Less than 5	101	30.89	300 people and below	197	60.24
6–10	112	34.25	300–3000 people	103	31.50
11–20	99	30.27	more than 3000 people	27	8.26
More than 20	15	4.59	Total	327	100
Total	327	100			

**Table 2:** Exploratory factor analysis results.

Factors and Items	EFA Loadings					
1. Work-Family Conflict (WFC)						
(1) Time-based work-family Conflict (TC)						
WFC 1 My work keeps me from my family activities more than I would like.	0.641					
WFC 2 The time I must devote to my job keeps me from participating equally in household responsibilities and activities.	0.761					
WFC 3 The time I spend with my family often causes me not to spend time at work activities that could be helpful to my career.	0.682					
WFC 4 The time I spend on family responsibilities often interferes with my work responsibilities.	0.528					
(2) Strain-based work-family Conflict (SC)						
WFC 5 I am often so emotionally drained when I get home from work that it prevents me from contributing to my family.		0.750				
WFC 6 Due to all the pressures at work, sometimes when I come home, I am too stressed to do the things I enjoy.		0.808				
WFC 7 Due to stress at home, I am often preoccupied with family matters at work.		0.587				
WFC 8 Tension and anxiety from my family life often weaken my ability to do my job.		0.607				
(3) Behavior-based work-family Conflict (BC)						
WFC 9 The problem-solving behaviors I use in my job are not effective in resolving problems at home.			0.832			
WFC 10 The behaviors that work for me at home do not seem to be effective at work.			0.832			
2. Perceived Organizational Support (POS)						
(1) Emotional Support (ES)						
POS 1 Help is available from my organization when I have problems supporting the elderly and children.				0.727		
POS 2 My organization really cares about my well-being.				0.802		
POS 3 My organization is willing to help me if I need a special favor at work.				0.789		
POS 4 My organization is willing to help me if I need a special favor in daily life.				0.830		
(2) Instrumental Support (IS)						
POS 5 My organization allows me work at home on family problems.					0.869	
POS 6 My organization allows me to work on my flex time subject to the approval.					0.825	
POS 7 The leave policy of my organization can meet my individual needs or demands from my family.					0.785	
3. Professional Commitment (PC)						
PC 2 I want the career I am doing now.						0.745
PC 3 If could do it all over, I would still choose my current career.						0.743
PC 4 If had all the money needed, I would still work in my current career.						0.735
PC 5 Ideal vocation too well to give it up.						0.785
% variance explained	39.45	11.99	9.58	48.20	21.05	56.60
Reliability	0.703	0.723	0.808	0.823	0.800	0.744

**Table 3:** Confirmatory factor analysis results.

Model	*χ*^2^/*df*	CFI	GFI	RMSEA	C.R.	AVE
1. Work-Family Conflict (WFC)					C.R._TC_ = 0.710 C.R._SC_ = 0.725 C.R._BC_ = 0.817	AVE_TC_ = 0.386 AVE_SC_ = 0.401 AVE_BC_ = 0.693
First-order, three-factor model (TC, SC, BC)	4.665	0.882	0.920	0.106		
Second-order factor model	4.665	0.882	0.920	0.106		
2. Perceived Organizational Support (POS)					C.R._ES_ = 0.827 C.R._IS_ = 0.805	AVE_ES_ = 0.550 AVE_IS_ = 0.581
First-order, two-factor model (ES, IS)	3.369	0.965	0.964	0.085		
Second-order factor model	3.369	0.965	0.964	0.085		
3. Professional Commitment (PC)					C.R._PC_ = 0.745	AVE_PC_ = 0.423
one-factor model	—	0.917	0.965	0.193		

Note: TC: Time-based work-family Conflict; SC: Strain-based work-family Conflict; BC: Behavior-based work-family Conflict; ES: Emotional Support; IS: Instrumental Support. CFI: Comparative Fit Index. GFI: Goodness of fit index. RMSEA: Root mean square error of approximation.

**Table 4:** The comparative results of alternative models.

Models Used to Discriminate the Measures	*χ*^2^/*df*	CFI	TLI	RMSEA	SRMR
Six-factor model: TC, SC, BC, ES, IS, PC	2.575	0.896	0.873	0.069	0.057
Five-factor model: TC, SC, BC, POS (ES *+* IS), PC	3.899	0.802	0.765	0.094	0.072
Four-factor model: WFC (TC *+* SC *+* BC), ES, IS, PC	3.610	0.818	0.789	0.089	0.066
Three-factor model: WFC (TC *+* SC *+* BC), POS (ES *+* IS), PC	4.826	0.728	0.691	0.108	0.079
One-factor model: all the factors merged	8.873	0.430	0.363	0.155	0.147

Note: WFC: Work-Family Conflict; TC: Time-based work-family Conflict; SC: Strain-based work-family Conflict; POS: Perceived Organizational Support; BC: Behavior-based work-family Conflict; ES: Emotional Support; IS: Instrumental Support; PC: Professional Commitment.

**Table 5:** Correlation analysis results.

	Mean	S.D.	TC	SC	BC	ES	IS	PC
TC	2.921	0.821	0.621					
SC	2.860	0.873	0.502 **	0.633				
BC	2.849	1.074	0.517 **	0.418 **	0.832			
ES	3.173	1.004	−0.089	−0.133 *	−0.105	0.742		
IS	3.325	0.849	−0.144 **	−0.180 **	−0.020	0.396 **	0.761	
PC	3.581	0.801	−0.188 **	−0.062	−0.150 **	0.566 **	0.387 **	0.650

Note: TC: Time-based work-family conflict; SC: Strain-based work-family conflict; BC: Behavior-based work-family conflict; ES: Emotional support; IS: Instrumental support; PC: Professional commitment; S.D.: Standard deviation. The square roots of AVE were reported in bold italic along the diagonal. ** *p* < 0.01; * *p* < 0.05; Two-tailed test.

**Table 6:** The results of independent samples *t*-test.

Variables	*t*-Test for Equality of Means			
	*t*	Significance	Lower 95% CI	Upper 95% CI
WFC	13.863	***	0.776	1.033
POS	−1.661	0.098	−0.326	0.027
PC	−1.791	0.075	−0.400	0.020

Notes: WFC: Work-family conflict; POS: Perceived organizational support; PC: Professional commitment. CI: Confidence interval. *** *p* < 0.001.

**Table 7:** The direct effects testing results.

Models		Unstandardized Estimate	Standardized Estimate	*p*-Value
Model 1	WFC→PC	−0.288	−0.250	**
		*χ*^2^/*df* = 3.552; CFI = 0.862; TLI = 0.828; RMSEA = 0.088; SRMR = 0.059.		
Model 2	TC→PC	−0.401	−0.378	*
	SC→PC	0.157	0.217	n.s.
	BC→PC	−0.050	−0.070	n.s.
	*χ*^2^/*df* = 3.579; CFI = 0.864; TLI = 0.826; RMSEA = 0.089; SRMR = 0.057.			
Model 3	POS→PC	0.815	0.818	***
		*χ*^2^/*df* = 3.014; CFI = 0.938; TLI = 0.917; RMSEA = 0.078; SRMR = 0.053.		
Model 4	ES→PC	0.525	0.616	***
	IS→PC	0.120	0.169	*
	*χ*^2^/*df* = 3.014; CFI = 0.938; TLI = 0.917; RMSEA = 0.078; SRMR = 0.053.			

Note: TC: Time-based work-family conflict; SC: Strain-based work-family conflict; BC: Behavior-based work-family conflict; ES: Emotional support; IS: Instrumental support; PC: Professional commitment. *** *p* < 0.001; ** *p* < 0.01; * *p* < 0.05; n.s., not significant. *χ*^2^, Chi-square; *df*, Degree of freedom; CFI: comparative fit index; TLI: Tucker-Lewis index; RMSEA: Root mean square error of approximation; SRMR: Standardized root mean square residual.

**Table 8:** The indirect effects testing results of the mediation model.

Model	Variables	Unstandardized Estimate	Standardized Estimate	Bootstrapping	
				Bias-Corrected 95% CI	
				Lower 2.5%	Upper 2.5%
Total Sample (*n* = 327)					
WFC-POS-PC	Influence paths				
	WFC→POS (a)	−0.247	−0.224	−0.524	−0.056
	POS→PC (b)	0.826	0.811	0.517	1.261
	WFC→PC	−0.054	−0.048	−0.252	0.190
	Indirect effect				
	a × b	−0.204	-	−0.509	−0.048
	*χ*^2^/*df* = 2.768; CFI = 0.870; TLI = 0.849; RMSEA = 0.074; SRMR = 0.062.				
Male Sample (*n* = 219)					
WFC-POS-PC	Influence paths				
	WFC→POS (a)	−0.696	−0.450	−1.214	−0.359
	POS→PC (b)	0.417	0.802	0.227	0.777
	WFC→PC	0.046	0.057	−0.199	0.265
	Indirect effect				
	a × b	−0.290	-	−0.645	−0.124
	*χ*^2^/*df* = 2.191; CFI = 0.856; TLI = 0.833; RMSEA = 0.074; SRMR = 0.073.				

Note: WFC: Work-family conflict; POS: Perceived organizational support; PC: Professional commitment. *χ*^2^: Chi-square; *df*: Degree of freedom; CFI: comparative fit index; TLI: Tucker-Lewis index; RMSEA: Root mean square error of approximation; SRMR: Standardized root mean square residual.

**Table 9:** The direct effects testing results of multiple mediation models.

Models	Variables	Unstandardized Estimate	Standardized Estimate	Bootstrapping	
				Bias-Corrected 95% CI	
				Lower 2.5%	Upper 2.5%
TC-POS-PC	Influence paths				
	TC→ES (a1)	−0.173	−0.148	−0.397	0.029
	TC→IS (a2)	−0.335	−0.229	−0.622	−0.115
	ES→PC (b1)	0.538	0.632	0.375	0.750
	IS→PC (b2)	0.120	0.176	−0.015	0.272
	TC→PC	−0.155	−0.156	−0.323	−0.004
	Indirect effect				
	a1 × b1	−0.093	-	−0.236	0.010
	a2 × b2	−0.040	-	−0.129	0.000
SC-POS-PC	Influence paths				
	SC→ES (a4)	−0.147	−0.187	−0.338	−0.014
	SC→IS (a5)	−0.281	−0.288	−0.446	−0.114
	ES→PC (b4)	0.539	0.658	0.369	0.751
	IS→PC (b5)	0.158	0.238	0.012	0.323
	SC→PC	0.065	0.101	−0.059	0.163
	Indirect effect				
	a4 × b4	−0.079	-	−0.205	−0.011
	a5 × b5	−0.044	-	−0.114	−0.009
BC-POS-PC	Influence paths				
	BC→ES (a7)	−0.107	−0.106	−0.241	0.033
	BC→IS (a8)	−0.068	−0.068	−0.229	0.149
	ES→PC (b7)	0.536	0.464	0.382	0.707
	IS→PC (b8)	0.164	0.141	0.015	0.307
	BC→PC	−0.115	−0.099	−0.302	0.006
	Indirect effect				
	a7 × b7	−0.057	-	−0.142	0.014
	a8 × b8	−0.011	-	−0.058	0.016

Note: TC: Time-based work-family conflict; SC: Strain-based work-family conflict; BC: Behavior-based work-family conflict; POS: Perceived organizational support; ES: Emotional support; IS: Instrumental support; PC: Professional commitment.

**Table 10:** Results of hypothesis testing.

Hypothesis	Coefficients	Conclusion
Hypothesis 1. Work-family conflict is negatively related to professional commitment of project employees.	−0.288 **	supported
Hypothesis 1a. TC→PC	−0.401 *	supported
Hypothesis 1b. SC→PC	0.157	not supported
Hypothesis 1c. BC→PC	−0.050	not supported
Hypothesis 2. Perceived organizational support is positively related to professional commitment of project employees.	0.815 ***	supported
Hypothesis 2a. ES→PC	0.525 **	supported
Hypothesis 2b. IS→PC	0.120 *	supported
Hypothesis 3. Work-family conflict has a negative indirect effect on professional commitment via perceived organizational support.	−0.204, 95% BC CI (−0.509; −0.048)	supported
Hypothesis 3a. TC-POS-PC	−0.093, 95% BC CI (−0.236; 0.010) (TC-ES-PC) −0.040, 95% BC CI (−0.129; 0.000) (TC-IS-PC)	not supported
Hypothesis 3b. SC-POS-PC	−0.079, 95% BC CI (−0.205; −0.011) (SC-ES-PC) −0.044, 95% BC CI (−0.114; −0.009) (SC-IS-PC)	supported
Hypothesis 3c. BC-POS-PC	−0.057, 95% BC CI (−0.142; 0.014) (BC-ES-PC) −0.011, 95% BC CI (−0.058; 0.016) (BC-IS-PC)	not supported

Notes: TC: Time-based work-family conflict; SC: Strain-based work-family conflict; BC: Behavior-based work-family conflict; POS: Perceived organizational support; ES: Emotional support; IS: Instrumental support; PC: Professional commitment. *** *p* < 0.001, ** *p* < 0.01, * *p* < 0.05 and 95% BC CI means 95% bias-corrected confidence interval.

## Appendix A

TITLE: THE MODEL OF WORK-FAMILY CONFLICT AND ACREER COMMITMENT
DATA:
FILE IS xxx.dat;
VARIABLE:
NAMES ARE WFC1 WFC2 WFC3 WFC4 WFC5 WFC6 WFC7 WFC8 WFC9 WFC10
POS1 POS2 POS3 POS4 POS5 POS6 POS7 POS8 POS9 POS10 PC2 PC3 PC4 PC5;
USEVARIABLES ARE WFC1 WFC2 WFC3 WFC4 WFC5 WFC6 WFC7 WFC8 WFC9 WFC10
PC2 PC3 PC4 PC5;
ANALYSIS:
ESTIMATOR = ML;
MODEL:
TC BY WFC1 WFC2 WFC3 WFC4;
SC BY WFC5 WFC6 WFC7 WFC8;
BC BY WFC9 WFC10;
WFC BY TC SC BC;
PC BY PC2 PC3 PC4 PC5;
PC ON WFC;
OUTPUT:
STANDARDIZED;

## Appendix B

TITLE: THE MEDIATION MODEL
DATA:
FILE IS xxx.dat;
VARIABLE:
NAMES ARE WFC1 WFC2 WFC3 WFC4 WFC5 WFC6 WFC7 WFC8 WFC9 WFC10
POS1 POS2 POS3 POS4 POS5 POS6 POS7 PC2 PC3 PC4 PC5;
USEVARIABLES ARE WFC1 WFC2 WFC3 WFC4 WFC5 WFC6 WFC7 WFC8
WFC9 WFC10 POS1 POS2 POS3 POS4 POS5 POS6 POS7 PC2 PC3 PC4 PC5;
ANALYSIS:
BOOTSTRAP = 1000;
MODEL:
TC BY WFC1 WFC2 WFC3 WFC4;
SC BY WFC5 WFC6 WFC7 WFC8;
BC BY WFC9 WFC10;
WFC BY TC SC BC;
ES BY POS1 POS2 POS3 POS4;
IS BY POS5 POS6 POS7;
POS BY ES IS;
PC BY PC2 PC3 PC4 PC5;
POS ON WFC (a);
PC ON WFC
POS (b);
MODEL CONSTRAINT:
NEW (ind);
ind = a*b;
OUTPUT:
STANDARDIZED;
SAMPSTAT;
CINTERVAL(BCBOOTSTRAP);
